# Impact of Quantum Non-Locality and Electronic Non-Ideality on the Shannon Entropy for Atomic States in Dense Plasma

**DOI:** 10.3390/e26070602

**Published:** 2024-07-16

**Authors:** Askhat T. Nuraly, Madina M. Seisembayeva, Karlygash N. Dzhumagulova, Erik O. Shalenov

**Affiliations:** 1 Department of General Physics, Satbayev University, Almaty 050013, Kazakhstan; askhat.nuraly.98@gmail.com (A.T.N.); seisembayevamm@gmail.com (M.M.S.); 2 Institute of Experimental and Theoretical Physics, Al-Farabi Kazakh National University, Almaty 050040, Kazakhstan; 3 Department of Plasma Physics, Nanotechnology and Computer Physics, Al-Farabi Kazakh National University, Almaty 050040, Kazakhstan

**Keywords:** Schrödinger equation, ground state, dense plasma, effective interaction potential, Shannon entropy

## Abstract

The influence of the collective and quantum effects on the Shannon information entropy for atomic states in dense nonideal plasma was investigated. The interaction potential, which takes into account the effect of quantum non-locality as well as electronic correlations, was used to solve the Schrödinger equation for the hydrogen atom. It is shown that taking into account ionic screening leads to an increase in entropy, while taking into account only electronic screening does not lead to significant changes.

## 1. Introduction

Dense non-ideal plasma appears in various astrophysical objects, such as the interiors of giant planets and White Dwarfs, as well as in laboratory conditions. High-energy-density studies have now received significant development due to the emergence of new high-tech and expensive devices, such as relativistic ion synchrotron accelerators, high-power lasers, intense charged particle beams, high-current Z-pinches, explosive and electroexplosive generators of powerful shock waves, and others. Temperature and density of dense non-ideal plasma are considered in the ranges $10^{4}–10^{6}\;K$ and $10^{21}–10^{25}\;{cm}^{-3}$, respectively.

At present, in the physics of dense non-ideal plasmas, the study of the influence of collective and quantum effects, such as screening, degeneracy, quantum non-locality, etc., on the properties of plasma is becoming increasingly relevant [1,2,3,4,5,6,7,8,9,10,11,12,13,14,15,16,17,18,19,20,21,22,23,24,25,26]. The results obtained in these studies are of crucial importance for expanding our knowledge of the physical processes in plasmas under extreme conditions, such as in the cores of stars, in experiments with laser plasmas, and in inertial thermonuclear fusion facilities [27].

In this paper, we investigate the influence of collective and quantum effects on the Shannon entropy of atomic states in quantum hydrogen plasma. Hydrogen plasma is one of the simplest and at the same time most important models for studying plasma systems. This is due to its wide applicability, ranging from astrophysical objects such as stars to laboratory setups used to study controlled thermonuclear fusion. Shannon entropy is a fundamental concept of information theory, introduced by Claude Shannon in 1948 to quantitatively describe the uncertainty in data transmission systems. The concept of Shannon entropy provides a unique tool for analyzing the complexity and orderliness of plasma states. In particular, visualizing entropy in spatial form allows one to clearly represent and quantify the degree of uncertainty and information content in the dynamics of plasma systems.

The work [28] considered the application of information entropy to the analysis of instabilities and turbulence in plasma. The study proposed methods for the quantitative assessment of chaos and order in plasma systems. L.C. Souza et al. [29] used Shannon entropy to study anomalous transport and chaotic processes in plasma. J.A. Krommes [30] applied the concept of information entropy to the modeling and analysis of structural changes in plasma, focusing on transitions between different regimes of the plasma state. G. Livadiotis [31] considered the use of Shannon entropy to analyze time series and spatial data obtained in plasma experiments, which revealed patterns and predicted the behavior of plasma structures. D. Rastovic [32] investigated the application of entropy in the context of thermonuclear fusion, proposing methods for improving the diagnostics and control of plasma in tokamak- and stellarator-type devices. These studies show that Shannon entropy is a powerful tool for analyzing and understanding complex processes in plasma.

Y.-D. Jung and M.-J. Lee [33,34,35] extensively studied Shannon entropy in the context of astrophysical plasma. In refs. [33,34], the effect of screening on the entropy of information states of atoms in strongly coupled plasma was analyzed. The studies showed that the change in the entropy of atomic states is more significant for excited states compared to the ground state and that the localization of screening has a greater effect on atoms with a higher charge number. In ref. [35], the influence of nonequilibrium effects on the Shannon entropy of atomic states in astrophysical Lorentz plasma was investigated. The study covered the calculation of the Shannon entropy for the ground and excited states of a hydrogen atom under astrophysical plasma conditions, taking into account the parameters of the spectral index, effective screening lengths, and plasma parameters, including radial and angular components. As a result of the study, it was found that the nonequilibrium nature of the Lorentz plasma suppresses the entropy changes in both the ground state and the excited states. It was also found that changes in entropy in excited states are more significant compared to the ground state. The effect of the magnetic quantum number on changes in entropy turned out to be insignificant, which is explained by the invariance of the angular parts of the Shannon entropy under the influence of the nonequilibrium nature and screening of the plasma.

The structure of the presented work is as follows: Section 2 describes the electron–ion interaction model for the calculation of the Schrödinger equation. The methodology of the calculation of the Shannon entropy is presented in Section 3. The fourth section presents the results obtained. Conclusions are given in Section 5.

## 2. Effective Potential of Electron–Ion Interaction

One of the main points in the study of phenomena in quantum plasma is the choice of an adequate model of particle interaction, taking into account various effects and features in this system. In ref. [26], the effective potential, which takes into account the effects of the quantum non-locality and electronic non-ideality, was presented. In this work, we use this electron–ion interaction potential (see Equation (6) from ref. [26]):

(1) $$\Phi_{ei}(r)=-\frac{Ze^{2}}{r\sqrt{(1+k_{i}^{2}\lambda_{ee}^{2}{)}^{2}-4k_{D}^{2}\lambda_{ee}^{2}}}\left(\left(1-\lambda_{ee}^{2}B^{2}\right)\exp(-Br)-\left(1-\lambda_{ee}^{2}A^{2}\right)\exp(-Ar)\right)$$

Here, $A^{2}=\frac{1+k_{i}^{2}\lambda_{ee}^{2}+\sqrt{(1+k_{i}^{2}\lambda_{ee}^{2}{)}^{2}-4k_{D}^{2}\lambda_{ee}^{2}}}{2\lambda_{ee}^{2}},B^{2}=\frac{1+k_{i}^{2}\lambda_{ee}^{2}-\sqrt{(1+k_{i}^{2}\lambda_{ee}^{2}{)}^{2}-4k_{D}^{2}\lambda_{ee}^{2}}}{2\lambda_{ee}^{2}}$, $\lambda_{ee}^{2}=\frac{{\tilde{a}}_{2}/{\tilde{a}}_{0}}{1-k_{Y}^{2}\gamma },$ ${\tilde{a}}_{0}=-2\pi e^{2}k_{Y}^{-2}$, ${\tilde{a}}_{2}=\frac{\hbar^{2}I_{-3/2}(\eta )}{36m_{e}n\theta^{\frac{3}{2}}I_{-1/2}^{2}(\eta )}$,$k_{D}^{2}=k_{s}^{2}+k_{i}^{2}=r_{D}^{-2},$ $k_{s}^{2}=\frac{k_{Y}^{2}}{1-k_{Y}^{2}\gamma },$ $k_{i}^{2}=\frac{4\pi n_{i}Z_{i}^{2}e^{2}}{k_{B}T_{i}}$ is the inverse ionic Debye length, $Z_{i}$ is the charge number of ions, parameter $\gamma =-\frac{1}{4\pi e^{2}}\frac{\partial^{2}\left[n_{e}f_{XC}\left(n_{e},T_{e}\right)\right]}{\partial n_{e}^{2}}$ takes into account the impact of electronic exchange correlations, $f_{XC}\left(n_{e},T_{e}\right)$ describes the exchange correlation part of the electronic free energy density from the quantum Monte Carlo simulations [26,36], $k_{Y}^{2}=\frac{1}{2}k_{TF}^{2}\theta^{1/2}I_{-1/2}(\eta )$ is the inverse electron screening length that interpolates between Debye and Thomas–Fermi expansions [26], $k_{TF}=\frac{\sqrt{3}\omega_{p}}{v_{F}}=\sqrt{\frac{4k_{F}}{\pi a_{B}}}$, $\omega_{p}=\sqrt{\frac{4\pi n_{e}e^{2}}{m_{e}}}$ is the plasma electronic frequency, and $v_{F}=\frac{\hbar k_{F}}{m_{e}}$ is the Fermi speed. Parameter $\theta =\frac{k_{B}T_{e}}{E_{F}}$ characterizes the electron degeneracy; here, $T_{e}$ is the electron temperature, $E_{F}=\frac{\hbar^{2}k_{F}^{2}}{2m_{e}}$ is the Fermi energy, $k_{F}={\left(3\pi^{2}n_{e}\right)}^{1/3}$ is the Fermi wave vector, ℏ is the reduced Planck constant, and $n_{e}$ and $m_{e}$ are the electron density and mass, respectively. In the work [26], the Schrödinger equation was solved on the basis of this model. As a result, wave functions and values of energy levels were obtained. In ref. [26], it was shown that if one takes $k_{i}=0$ in Equation (1), then the results of calculations of the energies of the ground and first excited levels of the hydrogen atom in plasma practically coincide with the data obtained by Z.-B. Chen et al. [37] based on the potential of Stanton and Murillo [38]. The potential of Stanton and Murillo takes into account only electron screening. A detailed description of the potential (1) with $k_{i}\neq 0$ is given in ref. [26]. 

The radial Schrödinger equation for a hydrogen atom in dense plasma would be given by

(2) $$\left[-\frac{\hbar }{2m_{e}}\left(\frac{d^{2}}{dr^{2}}-\frac{l(l+1)}{r^{2}}\right)+\Phi_{ei}(r)\right]P_{nl}(r)=E_{nl}P_{nl}(r),$$

where $P_{nl}(r)$ is the radial wave function for the *nl*th shell, $E_{nl}$ is the energy level, $\Phi_{ei}(r)$ is the corresponding electron–ion interaction potential, and r is the radius of the electron orbit. We used potential (1) as the interaction potential. Solving the Schrödinger equation based on interaction potentials in dense plasma is very difficult. Therefore, we used the variational method to calculate the bound states and wave functions of the hydrogen atom.

Using the variational method, we selected the trial wave functions as follows:

(3) $$P_{nl}(r)={rR}_{nl}(r)=-\sqrt{{\left(\frac{2Z}{n\alpha }\right)}^{3}\frac{\left(n-l-1\right)!}{2n{\left[(n+l)!\right]}^{3}}}e^{-\frac{\rho }{2}}\rho^{l}L_{n+l}^{2l+1}(\rho ),$$

where $\rho =\frac{2Z}{n\alpha }r$, α is the variational parameter, $\alpha \to a_{z}$ for $k_{D}\to 0$ & $\lambda_{ee}\to 0$, $a_{z}=a_{0}/Z$, $a_{0}$ is the Bohr radius, and $k_{D}\to 0$ & $\lambda_{ee}\to 0$ indicates a plasma-free situation. $L_{n+l}^{2l+1}(\rho )$ is the generalized Laguerre polynomials. The formula for the Laguerre polynomials was as follows:

(4) $$L_{n+l}^{2l+1}(\rho )=\sum_{i=0}^{n-l-1}{\left(-1\right)}^{i+1}\frac{{\left[\left(n+l\right)!\right]}^{2}}{(n-l-i-1)(2l+i+1)!i!}\rho^{i}.$$

The trial functions for the ground state 1s and the excited states 2s, 2p of the hydrogen atom were as follows:

(5) $$P_{1s}(r)=2\alpha^{-3/2}re^{-r/\alpha },\;P_{2s}(r)=\frac{1}{\sqrt{2}}\alpha^{-3/2}r\left(1-\frac{r}{2\alpha }\right)e^{-r/2\alpha },\;P_{3s}(r)=\frac{2}{3\sqrt{3}}\alpha^{-3/2}r\left(1-\frac{2r}{3\alpha }+\frac{2r^{2}}{27\alpha^{2}}\right)e^{-r/3\alpha },\;P_{2p}(r)=\frac{1}{2\sqrt{6\alpha^{5}}}r^{2}e^{-r/2\alpha },\;P_{3p}(r)=\frac{4\sqrt{2}}{9\sqrt{3\alpha^{5}}}r^{2}\left(1-\frac{r}{6\alpha }\right)e^{-r/3\alpha }.$$

The dependence of $\left\langle E_{nl}(\alpha )\right\rangle$ was found through the trial functions:

(6) $$\left\langle E_{nl}(\alpha )\right\rangle =\frac{\hbar^{2}}{2m}\int_{0}^{\infty }\left\{{\left(\frac{dP_{nl}(r)}{dr}\right)}^{2}+\left[\frac{l(l+1)}{r^{2}}+\frac{2m}{\hbar^{2}}\Phi_{ei}(r)\right]{P_{nl}}^{2}(r)\right\}dr.$$

The minima of this function gave the energy levels, and by substituting the values of $\alpha_{min}$ into the trial functions, the radial wave functions were determined.

In present work, we recalculated the Schrödinger equation on the basis of the Shalenov–Nuraly–Dzhumagulova potential (1) at $k_{i}^{\;}\;\neq \;0$ (the SNDP-A case) and potential Shalenov–Nuraly–Dzhumagulova (1) at $k_{i}^{\;}\;\neq \;0$ (SNDP-B case) in order to investigate the Shannon entropy of the system under study.

## 3. Methodology

It was shown that the Shannon entropy associated with the atomic density distribution $\rho (r)=|\psi (r){|}^{2}$ in the position space is defined as

(7) $$S_{\rho }=-\int \rho (r)\ln\rho (r)dr^{3}=-\int |\psi (r){|}^{2}\ln|\psi (r){|}^{2}dr^{3},$$

where ψ(r) is the atomic wave function in the position space r. In one-electron systems with the wave function

$\psi (r)=R_{nl}(r)Y_{lm}(\Omega )$, the Shannon entropy $S_{\rho }$ would be decomposed into the radial and angular parts [39]:

(8) $$S_{\rho }=S(R_{nl})+S(Y_{lm}),$$

where $S(R_{nl})$ is the radial part of the Shannon entropy with the radial wave function $R_{nl}$:

(9) $$S(R_{nl})=-\int {\left|R_{nl}\right|}^{2}\ln|R_{nl}{|}^{2}r^{2}dr,$$

$S(Y_{lm})$ is the angular part of the Shannon entropy with the spherical harmonics $Y_{lm}(\Omega )$:

(10) $$S(Y_{lm})=-\int |Y_{lm}(\Omega ){|}^{2}\ln|Y_{lm}(\Omega ){|}^{2}d\Omega ,$$

and dΩ=(sinθdθdφ) is the differential solid angle in spherical coordinates.

## 4. Results and Discussions

**Figure 1:**
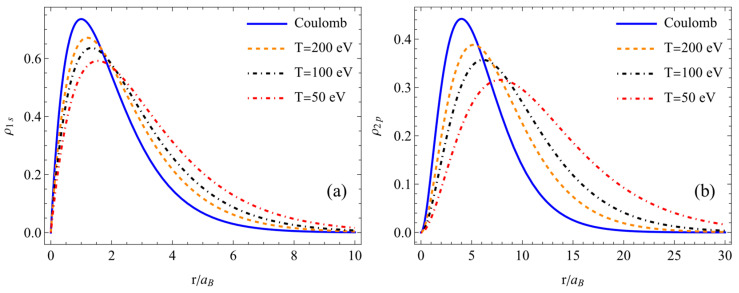
Atomic density distribution of the hydrogen atom in the ground state (**a**), $n_{e}=10^{24}\;{cm}^{-3}$, and excited state (**b**), $n_{e}=10^{23}\;{cm}^{-3}$.

**Figure 2:**
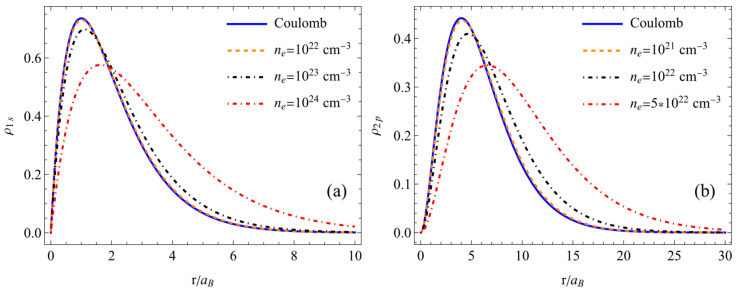
Atomic density distribution of the hydrogen atom in the ground state (**a**) and excited state (**b**), T = 40 eV.

The results calculated from the atomic density distribution of the hydrogen atom in the ground state (1s) in Figure 1a and Figure 2a and the excited state (2p) in Figure 1b and Figure 2b are given. The atomic density distributions were determined for different temperatures at a fixed plasma concentration in Figure 1 and different concentrations at a fixed plasma temperature in Figure 2. What can be observed from these figures is that as the temperature decreased and the concentration increased, the atomic density distribution decreased and spread out in space owing to less nuclear attraction felt due to screening by free electrons and quantum nonlocality. Conversely, as the temperature increased and the concentration decreased, the results approached the values of the atomic density distribution determined based on the Coulomb potential.

**Figure 3:**
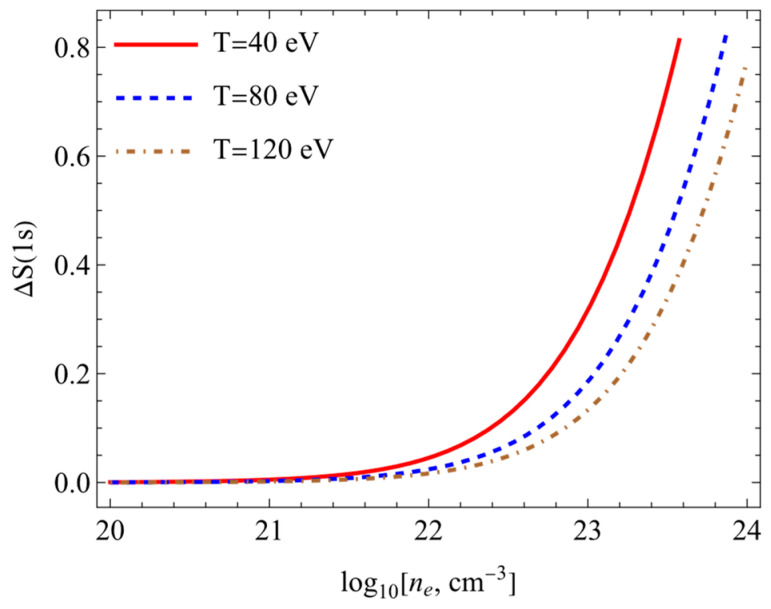
Entropy changes $\Delta S\left(1s\right)$ plotted as functions of the electron numerical density at different temperatures.

**Figure 4:**
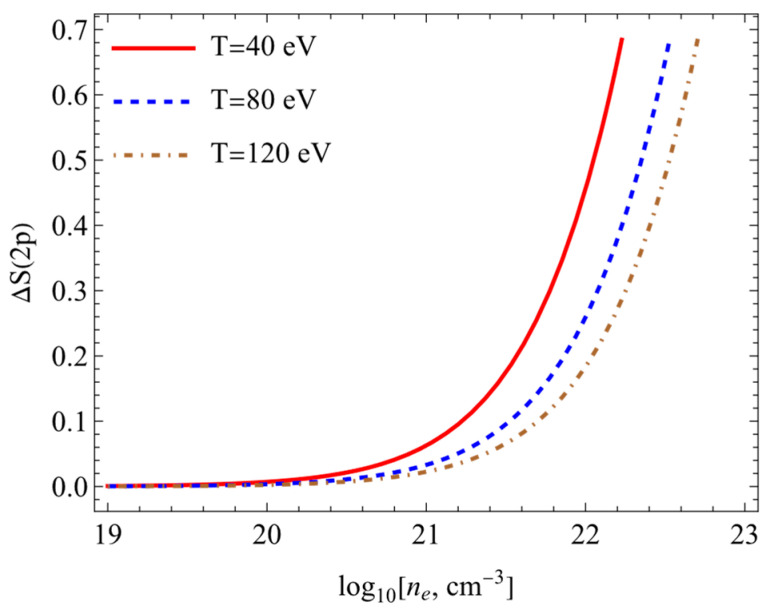
Entropy changes $\Delta S\left(2p\right)$ plotted as functions of the electron numerical density at different temperatures.

**Figure 5:**
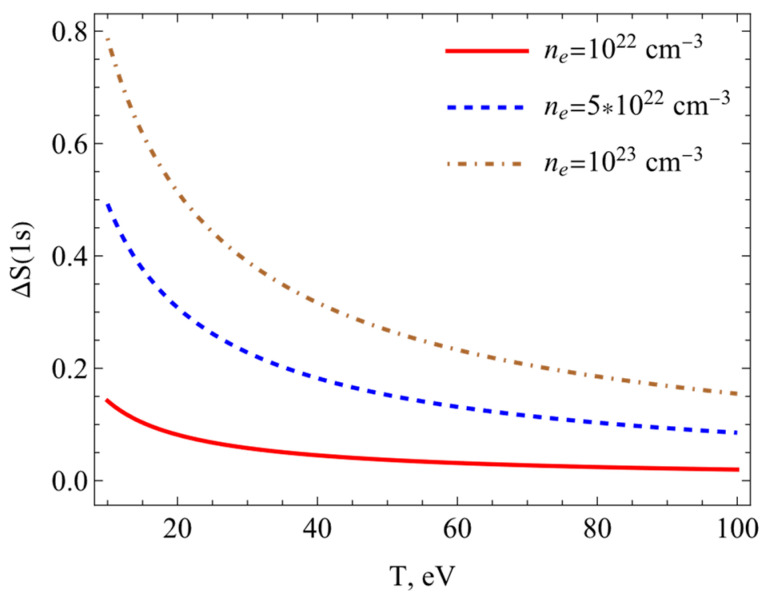
Entropy changes $\Delta S\left(1s\right)$ plotted as a function of the temperature at different electron numerical densities.

**Figure 6:**
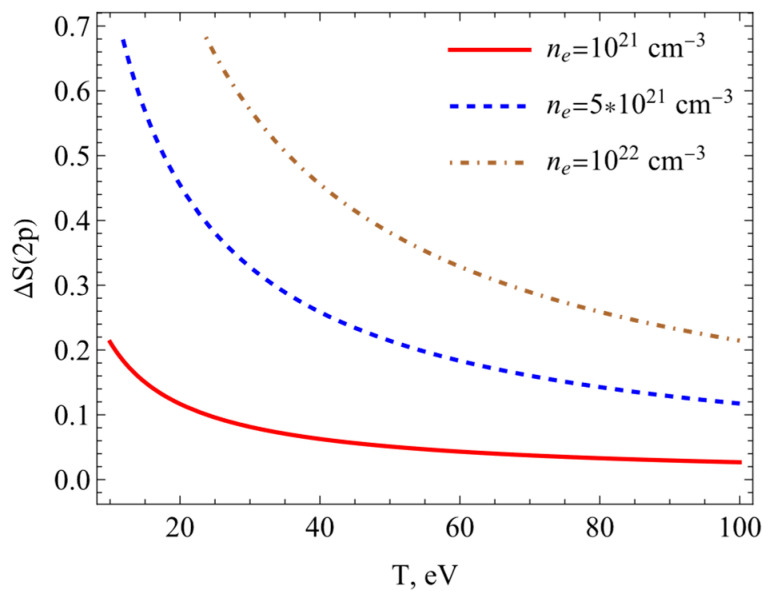
Entropy changes $\Delta S\left(2p\right)$ plotted as a function of the temperature at different electron numerical densities.

The results of calculations of the Shannon information entropy (7) are presented in this section. Figure 3, Figure 4, Figure 5 and Figure 6 show the changes in the Shannon information entropy ($\Delta S=S_{\rho }^{plasma}-S_{\rho }^{free}$). $S_{\rho }^{plasma}$ corresponds to the calculations of the Shannon information entropy (7) using potential (1) (the SNDP-B case). $S_{\rho }^{free}$ presents data obtained on the basis of the Coulomb potential for the isolated atom. One can see from Figure 3, Figure 4, Figure 5 and Figure 6 that the entropy changes fell with an increase in the temperature and rose with an increase in the electron numerical density. Figure 3 and Figure 4 show the changes in Shannon information entropy (ΔS) as functions of the electron numerical density for the 1s state (Figure 3) and 2p state (Figure 4). The changes in Shannon information entropy as functions of the temperature are presented on Figure 5 (1s state) and Figure 6 (2p state). Also, one can see that the entropy change in excited states was more prominent than that in the ground state. This fact was mentioned in refs. [4,33,34,35,40,41].

**Figure 7:**
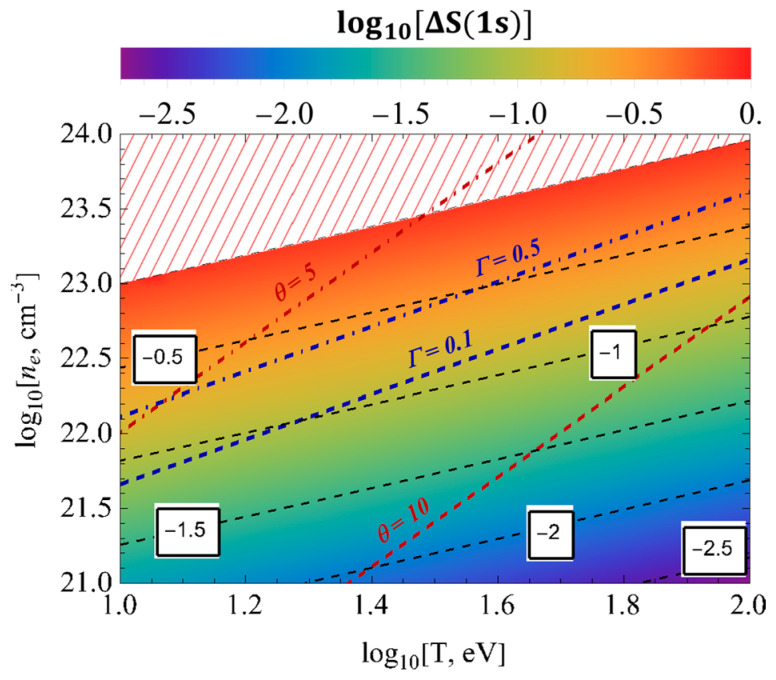
The surface plots of the entropy changes $\Delta S\left(1s\right)$ as a function of the temperature and electron numerical densities.

**Figure 8:**
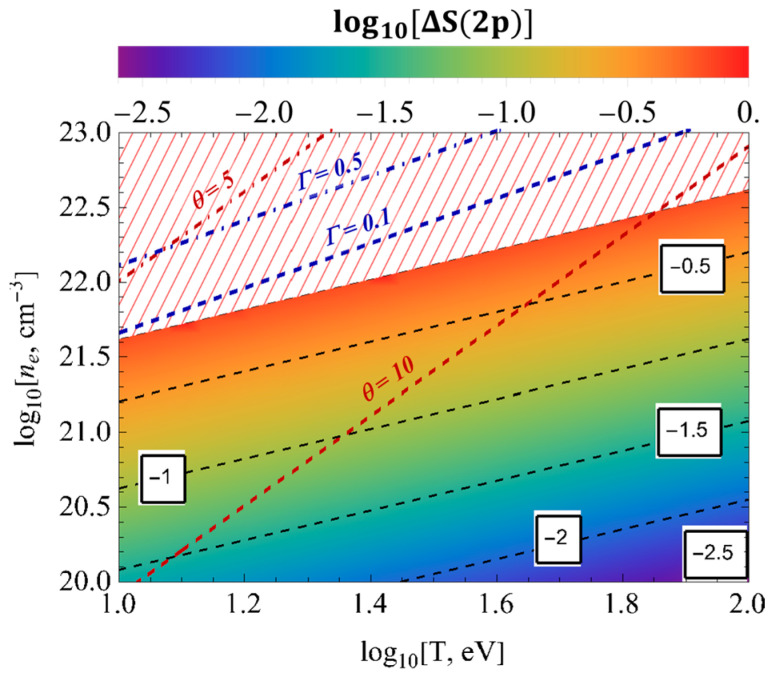
The surface plots of the entropy changes $\Delta S\left(2p\right)$ as a function of the temperature and electron numerical densities.

Figure 7 and Figure 8 represent the surface plots of the entropy changes $\Delta S\left(1s\right)$ and $\Delta S\left(2p\right)$, respectively, as a function of the temperature and electron numerical densities. These figures show that the influence of screening effects, quantum non-locality, and electronic correlations for the entropy changes $\Delta S\left(1s\right)$ and $\Delta S\left(2p\right)$ in the dense cold plasmas was more pronounced than in the rarefied hot plasmas.

Table 1 and Table 2 present the general behavior of the absolute change in Shannon entropies for hydrogen plasma $\Delta S\left(1s\right)$ (Table 1) and $\Delta S\left(2p\right)$ (Table 2) of an atom immersed in plasma compared to a free atom, i.e., $S=S^{plasma}-S^{free}$. The calculations were performed for four values of the temperature at four values of electron number densities. We performed the calculations and present the results based on the following models: with the Debye–Huckel potential (DHP), with the Shalenov–Nuraly–Dzhumagulova potential (1) at $k_{i}^{\;}\;\neq \;0$ (SNDP-A), and, finally, with the Shalenov–Nurala–Dzhumagulova potential (1) at $k_{i}^{\;}\;\neq \;0$; (SNDP-B). The results corresponding to $n_{e}=10^{22}\;{cm}^{-3}$ were compared with the data of ref. [35], where the DHP was used.

As one can see, our DHP data were in good agreement with those obtained in ref. [35]. The results for SNDP-1 were slightly less than for DHP but the results for SNDP-B were approximately twice as large as the other results. This means that taking into account ionic screening leads to an increase in entropy, while taking into account only electronic screening does not lead to significant changes.

The results for the radial Shannon entropy S ($R_{nl}$) (see Equation (9)) for hydrogen plasma for quantum numbers n≤3 and l=0,1 are presented in Table 3. The variation of the radial Shannon entropy with respect to *n* and *l* for free hydrogen was previously discussed by Jiao et al. [39] and Yanez et al. [42]. Here, it can be seen that our calculations for the isolated atom case (without plasma) were in excellent agreement with the data obtained in [39]. When we took into account the plasma effects based on potential (1) (the SNDP-B case), the results showed the same conclusions that we made based on Figure 1, Figure 2, Figure 3 and Figure 4.

## 5. Conclusions

In this paper, we investigated the effect of quantum non-locality as well as electronic correlations on the variation of the Shannon entropy for atomic states in dense plasmas. The results obtained were verified by comparison with data obtained by other authors for isolated atoms and atoms immersed in plasma at certain values of plasma parameters. In all cases, excellent agreement was noted.

The influence of temperature and electron density on the Shannon information entropy of atomic states in dense plasma was studied. It was concluded that quantum non-locality and electronic correlations are crucial for accurately describing entropy and atomic state distribution in dense plasmas. Therefore, Shannon atomic entropy can provide information about atomic states and plasma parameters, such as density and temperature. It would be useful in plasma diagnostics, as Shannon atomic entropy measures the transmission of quantum information [43] during atomic collisions and radiation processes depending on plasma conditions. These findings are significant for understanding dense plasmas in astrophysical objects and high-energy-density laboratory conditions, providing insights for controlled thermonuclear fusion, laser–plasma experiments, and the study of stellar interiors.

## Figures and Tables

**Table 1:** Shannon entropy changes $\Delta S\left(1s\right)$ for hydrogen plasma compared with the results of ref. [35]. DHP: present work with Debye–Huckel potential ($\lambda_{ee}=0$, $k_{i}^{\;}=0$); SNDP-A: present work with Shalenov–Nuraly–Dzhumagulov potential (1),$\;k_{i}^{\;}=0$; SNDP-B: present work with Shalenov–Nuraly–Dzhumagulov potential (1), $k_{i}\;\neq \;0$.

T,[eV]	Model	$lg(n_{e},[{cm}^{-3}])$			
		21	22	23	24
50.5	DHP	0.002156	0.019815 0.019826 ^a^	0.155426	0.830312
	SNDP-A	0.002051	0.018773	0.145797	0.773562
	SNDP-B	0.004134	0.036685	0.265969	-
202	DHP	0.000550	0.005263 0.005272 ^b^	0.046196	0.325082
	SNDP-A	0.000542	0.005186	0.045446	0.318487
	SNDP-B	0.001083	0.010180	0.085225	0.545540
454.5	DHP	0.000246	0.002389 0.002393 ^c^	0.021843	0.170048
	SNDP-A	0.000244	0.002373	0.021687	0.168575
	SNDP-B	0.000488	0.004679	0.041358	0.297958
808	DHP	0.000139	0.001358 0.001361 ^d^	0.012687	0.104340
	SNDP-A	0.000138	0.001353	0.012636	0.103853
	SNDP-B	0.000276	0.002675	0.024341	0.187644

^a^ Ref. [35], $r_{D}=10a_{B}$; ^b^ ref. [35], $r_{D}=20a_{B}$; ^c^ ref. [35], $r_{D}=30a_{B}$; ^d^ ref. [35], $r_{D}=40a_{B}$.

**Table 2:** Shannon entropy changes $\Delta S\left(2p\right)$ for hydrogen plasma compared with the results of ref. [35]. DHP: present work with Debye–Huckel potential ($\lambda_{ee}=0$, $k_{i}^{\;}=0)$; SNDP-A: present work with Shalenov–Nuraly–Dzhumagulova potential (1),$\;k_{i}^{\;}=0$; SNDP-B: present work with Shalenov–Nuraly–Dzhumagulova potential (1), $k_{i}^{\;}\neq \;0$.

T,[eV]	Model	$lg(n_{e},[{cm}^{-3}])$			
		20	21	22	23
50.5	DHP	0.002879	0.026589	0.213152 0.213257 ^a^	-
	SNDP-A	0.002866	0.026457	0.211904	-
	SNDP-B	0.005655	0.050643	0.378147	-
202	DHP	0.000733	0.007040	0.062325 0.062422 ^b^	0.453349
	SNDP-A	0.000732	0.007032	0.062243	0.452619
	SNDP-B	0.001455	0.013732	0.116375	-
454.5	DHP	0.000328	0.003192	0.029345 0.029395 ^c^	0.232816
	SNDP-A	0.000328	0.003190	0.029328	0.232663
	SNDP-B	0.000652	0.006277	0.055917	0.412632
808	DHP	0.000185	0.001814	0.017010 0.017040 ^d^	0.141772
	SNDP-A	0.000185	0.001813	0.017004	0.141723
	SNDP-B	0.000368	0.003582	0.032771	0.257123

^a^ Ref. [35], $r_{D}=10a_{B}$; ^b^ ref. [35], $r_{D}=20a_{B}$; ^c^ ref. [35], $r_{D}=30a_{B}$; ^d^ ref. [35], $r_{D}=40a_{B}$.

**Table 3:** Radial Shannon entropy $S_{\rho }$ for hydrogen plasma for quantum numbers n≤3 and l=0,1. PFJ: plasma-free, Jiao et al. [39]; PFPW: plasma-free, present work.

Plasma Type	$lg(n_{e},\left[{cm}^{-3}\right])$	1s	2s	3s	2p	3p
**PFJ**	-	1.61371	5.57991	7.89546	5.16582	7.70677
**PFPW**	-	1.61371	5.57999	7.89596	5.16589	7.70723
T=20 eV	19	1.61381	5.58165	7.90401	5.16728	7.71472
	20	1.61475	5.59627	7.96978	5.17961	7.77645
	21	1.62353	5.71449	8.40644	5.28263	8.19733
T=10 eV	19	1.61391	5.58335	7.91208	5.16869	7.72225
	20	1.61571	5.61123	8.03277	5.19235	7.83611
	21	1.63199	5.82120	8.73711	5.37814	8.52553
T=5 eV	19	1.61410	5.58662	7.92741	5.17142	7.73658
	20	1.61746	5.63889	8.14261	5.21599	7.94099
	21	1.64678	5.99741	-	5.53953	-

## Data Availability

The data that support the findings of this study are available from the corresponding author upon reasonable request.
